# An intensivist-led tracheostomy review team is associated with shorter decannulation time and length of stay: a prospective cohort study

**DOI:** 10.1186/cc6864

**Published:** 2008-04-11

**Authors:** Antony E Tobin, John D Santamaria

**Affiliations:** 1 Intensive Care Unit, St. Vincent's Hospital Melbourne, PO Box 2900, Fitzroy VIC 3065, Australia; 2 University of Melbourne, Victoria 3010 Australia

## Abstract

**Introduction:**

Without specific strategies to address tracheostomy care on the wards, patients discharged from the intensive care unit (ICU) with a tracheostomy may receive suboptimal care. We formed an intensivist-led multidisciplinary team to oversee ward management of such patients. To evaluate the service, we compared outcomes for the first 3 years of the service with those in the year preceding the service.

**Methods:**

Data were prospectively collected over the course of 3 years on ICU patients not under the care of the ear, nose, and throat unit who were discharged to the ward with a tracheostomy and compared with outcomes in the year preceding the introduction of the service. Principal outcomes were decannulation time, length of stay after ICU discharge, and stay of less than 43 days (upper trim point for the disease-related group [DRG] for tracheostomy). Analysis included trend by year and multivariable analysis using a Cox proportional hazards model. *P *values of less than 0.05 were assumed to indicate statistical significance. As this was a quality assurance project, ethics approval was not required.

**Results:**

Two hundred eighty patients were discharged with a tracheostomy over the course of a 4-year period: 41 in 2003, 60 in 2004, 95 in 2005, and 84 in 2006. Mean age was 61.8 (13.1) years, 176 (62.9%) were male, and mean APACHE (Acute Physiology and Chronic Health Evaluation) II score was 20.4 (6.4). Length of stay after ICU decreased over time (30 [13 to 52] versus 19 [10 to 34] days; *P *< 0.05 for trend), and a higher proportion of decannulated patients were discharged under the upper DRG trim point of 43 days (48% versus 66%; *P *< 0.05). Time to decannulation after ICU discharge decreased (14 [7 to 31] versus 7 [3 to 17] days; *P *< 0.01 for trend). Multivariate analysis showed that the hazard for decannulation increased by 24% (3% to 49%) per year.

**Conclusion:**

An intensivist-led tracheostomy team is associated with shorter decannulation time and length of stay which may result in financial savings for institutions.

## Introduction

Tracheostomy in the intensive care unit (ICU) is increasingly used as a means to speed weaning from mechanical ventilation and to provide a safe airway [1]. Tracheostomy allows earlier discharge of patients from the ICU, thus allowing better management of limited ICU resources [2,3], and may be associated with reduced mortality [4,5]. The advent of percutaneous tracheostomy has meant that surgical teams are increasingly divorced from the tracheostomy management of ICU patients [1,6]. As a result, patients may be discharged to the wards with tracheostomies but without links to surgical teams that traditionally managed ward tracheostomies. Without specific strategies to address tracheostomy care on the wards, such patients may potentially receive suboptimal care. Clec'h and colleagues [7] reported that ICU patients who received tracheostomies and were sent to the ward from the ICU with a tracheostomy *in situ *had significantly higher odds of death than those patients decannulated in the ICU prior to discharge. Poor tracheostomy care on the wards was one explanation suggested for this difference.

At our institution, prior to 2004, physiotherapists and speech pathologists oversaw tracheostomy weaning of all patients not under the ear, nose, and throat (ENT) unit bedcard with *ad hoc *input from doctors. Specialist input from the ICU or the ENT service was on an individual case referral basis and as a result specialist input was inconsistent and often delayed. Review of outcomes for such patients in the ICU mortality and morbidity meetings noted that there were numerous medical emergency team (MET) calls for hypoxia and 'threatened airway' amongst ICU patients discharged to the ward with a tracheostomy. On review, it was felt that one patient had died due to occlusion of his tracheostomy and that this may have been preventable. This led to the formation of an intensivist-led multidisciplinary team to oversee the management of all patients discharged to the ward from the ICU with a tracheostomy *in situ *who were not under the ENT bedcard.

At the initiation of the service, a database was created to prospectively collect information on outcomes felt to be relevant for demonstrating the impact of the team on patient care. Our *a priori *hypothesis was that tracheostomy care provided by an intensivist-led multidisciplinary team would shorten decannulation time and reduce post-ICU hospital length of stay compared with the old model of *ad hoc *tracheostomy care. This paper reports on these outcomes for the first 3 years of the service as well as baseline data from the year prior to the service's inception.

## Materials and methods

St. Vincent's Hospital Melbourne is a 400-bed tertiary referral hospital associated with the University of Melbourne, Australia. There is a single ICU in the hospital and it receives 1,100 to 1,200 admissions per year, of which approximately 40% are cardiac surgical cases. There are 10 general beds and 2 cardiac surgical beds, and the median and average lengths of stay are 26.5 (19.5 to 70.5) hours and 69.6 (105.1) hours, respectively. All tracheostomy patients discharged from the ICU alive who were not under the ENT unit's care were followed up on the wards by the multidisciplinary tracheostomy review team.

The team consists of an intensivist, an ICU liaison nurse, a physiotherapist, a speech pathologist, and a dietician. Twice-weekly ward rounds are performed to review patients and to plan and oversee an individualised tracheostomy weaning programme. A bedside assessment is made of the patient's ability to tolerate cuff deflation; upper airway patency; and speech, cough, and oxygen requirements. From this, an individualised plan for cuff deflation trials, use of speaking valves, and swallowing assessments is made. In addition, a bed area check is made to ensure that humidifiers and suction are set up correctly and working and that spare tracheostomy tubes of the same size and one size smaller and tracheal dilators are at the bedside.

Patients are decannulated when they are tolerating 24-hour cuff deflation, have a patent upper airway (as demonstrated by speech with a Passey-Muir valve or the ability to tolerate tracheostomy tube occlusion), and are able to clear respiratory secretions via the mouth without the need for suctioning. These general criteria are adjusted according to specific patient situations and other ongoing medical problems and interventions. Because of reduced specialist services on weekends, patients generally are not decannulated on Fridays. Tracheostomy tubes are not changed routinely but only when downsizing is felt to be necessary for weaning or when cuff or tube patency is problematic. Tubes without inner cannulas are used routinely, although tubes with inner cannulas are used if secretions are thick and compromise tube patency. All patients receive heated humidification.

Within normal working hours, the ICU liaison nurse and the intensivist are available to review patients or address problems encountered by ward nurses or allied medical staff. Out of hours, the ICU provides any necessary assistance for acute problems either by direct consultation or via the MET/cardiac arrest teams that are run in conjunction with the ICU. In addition to patient care, the team is responsible for drafting and updating the hospital's ward tracheostomy protocol and the ICU liaison nurse provides regular tracheostomy education sessions for ward nurses.

Data were collected prospectively and stored in the ICU database. Demographics, hospital and ICU admission and discharge times, Acute Physiology and Chronic Health Evaluation II (APACHE II) score on admission, admission unit, indication for tracheostomy, time from ICU discharge to decannulation, and discharge destination were recorded. Admitting units were categorised as medical, cardiothoracic, neurosurgical, or other surgical with medical as base. Indication for tracheostomy was categorised as prolonged ventilation/weaning, coma, failed extubation, and other (includes post-extubation stridor and difficult airway) with prolonged ventilation/weaning as base. For patients who had more than one ICU admission during their hospital stay, the ICU admission during which the tracheostomy was inserted was used for data analysis. To be able to include baseline data prior to the institution of the service, the ICU patient database was searched for patients who had a tracheostomy whilst in the ICU in 2003. The medical records for these patients were retrieved and data on decannulation time from ICU discharge were extracted and combined with data from the ICU database to provide a dataset with most of the elements of the prospectively collected one. As this was a quality review project, ethics approval was not required.

The primary outcome measure was decannulation time from ICU discharge. Secondary outcome measures of interest were hospital length of stay, length of stay after ICU discharge, and length of stay of less than 43 days (the upper trim point for the disease-related group [DRG] code for tracheostomy).

For continuous variables, results are expressed as mean (standard deviation) or median (interquartile range), depending on the normality of distribution. Number and percentage are reported for categorical variables. Univariate analyses include Kruskal-Wallis test for continuous variables and chi-square or Fisher's exact test for categorical variables. Trend over time was examined using Cuzick's test for trend for continuous variables and the chi-square trend test for categorical variables. Kaplan-Meier survival curves for decannulation times were compared with the log-rank test. Multivariable analysis of decannulation times was undertaken using a Cox proportional hazards model. The proportional hazards assumption was inspected graphically and tested statistically. Hazard ratios are presented with 95% confidence intervals. A *P *value of less than 0.05 was assumed to indicate statistical significance. Analyses were performed with STATA version 9.2 (StataCorp LP, College Station, TX, USA).

## Results

**Figure 1:**
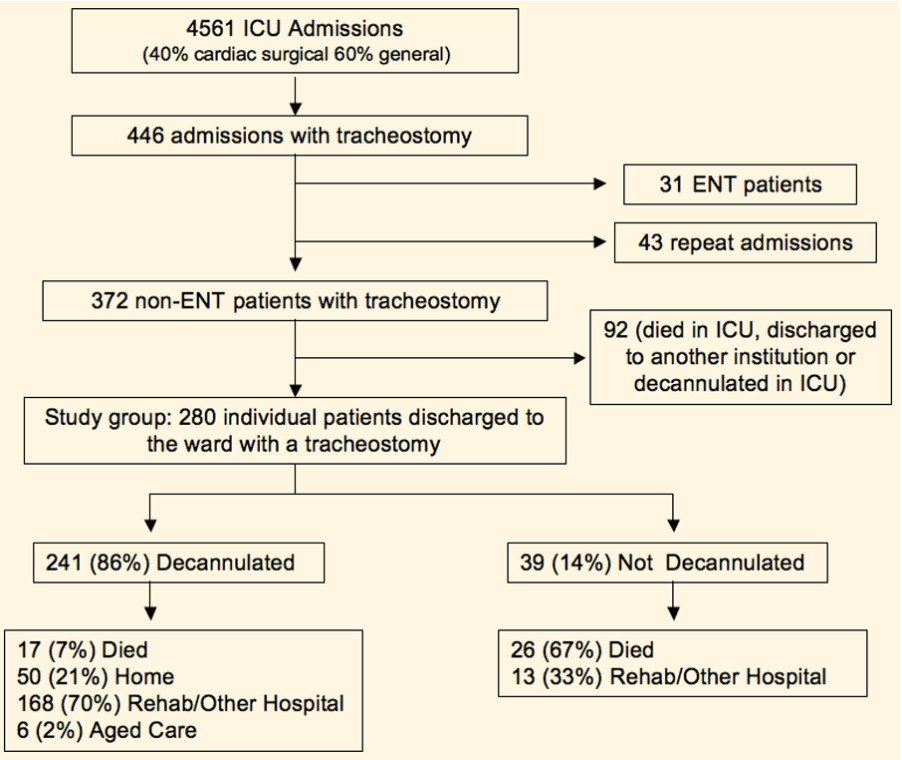
Patient flowchart. ENT, ear, nose, and throat; ICU, intensive care unit.

Four thousand five hundred sixty-one admissions occurred over the course of the 4-year period (Figure 1) with 280 individual patients discharged to the wards from the ICU with a tracheostomy: 41 in 2003, 60 in 2004, 95 in 2005, and 84 in 2006. Eight patients were discharged to the ward while requiring nocturnal ventilatory support that was subsequently weaned. Overall, 37 patients were readmitted to the ICU: 31 once and 6 twice. Three patients were readmitted to the ICU after decannulation: one following new sepsis, one following an operative procedure, and the other following a myocardial infarct. All three had tracheostomies reinserted. For these three patients only, the subsequent tracheostomy and ICU admission were included in the study as it was felt that it was most likely to be the one that influenced hospital outcome.

The mean age was 61.8 (13.1) years, 176 (62.9%) were male, and the mean APACHE II score was 20.4 (6.4) (Table 1). The major indications for tracheostomy were prolonged ventilation/weaning (58%) and coma (21%), with no difference evident between the years. The mix of patients by admitting unit was similar across the years although the proportion of cardiac surgical patients has increased over time. Intensivists inserted the majority of tracheostomies, with the proportion of surgical tracheostomies declining over the study period (*P *< 0.05). Of the 280 patients, 241 (86%) were decannulated prior to discharge, of whom 17 (7%) died, 50 (21%) were discharged home, 168 (70%) were discharged to a rehabilitation unit or another hospital, and 6 (2%) were discharged to aged care. Of the 39 (14%) not decannulated, 26 (67%) died and 13 (33%) were discharged to a rehabilitation unit or another hospital (Figure 1). Mortality decreased over the years but the trend was not statistically significant (*P *= 0.1) (Table 1).

**Table 1:** Patient demographics

	All	2003	2004	2005	2006
ICU admissions	4,561	1,169	1,146	1,128	1,119
ICU tracheostomy	415	70	83	128	134
Discharged with tracheostomy	280	41	60	95	84
					
Age in years, mean (SD)	61.8 (13.2)	58.7 (13.6)	62.2 (13.1)	62.6 (13.7)	62.1 (12.4)
Male	176 (63)	26 (63)	42 (70)	59 (62)	49 (58)
APACHE II score, mean (SD)	20.4 (6.4)	21.7 (7.1)	20.3 (6.1)	20.3 (6.0)	20.1 (6.5)
Admitting unit					
Neurosurgery	65 (23)	12 (29)	15 (25)	21 (22)	17 (20)
Cardiothoracic	71 (25)	1 (2)	15 (25)	21 (22)	34 (41)
Surgery	38 (14)	4 (10)	11 (18)	16 (17)	7 (8)
Medical	106 (40)	24 (58)	19 (32)	37 (39)	26 (31)
Indication					
Prolonged ventilation	138 (58)	-	34 (57)	52 (55)	52 (62)
Coma	51 (21)	-	13 (22)	19 (19)	19 (23)
Failed extubation	29 (12)	-	5 (8)	18 (19)	6 (7)
Other	21 (9)	-	8 (13)	6 (6)	7 (8)
Method					
Surgical	43 (15)	10 (24)	14 (23)	11 (12)	8 (10)
Percutaneous	237 (85)	31 (76)	46 (77)	84 (88)	76 (90)
Discharge destination					
Home	50 (18)	4 (10)	8 (13)	17 (18)	21 (25)
Other hospital	111 (40)	20 (49)	25 (42)	40 (42)	26 (31)
Rehabilitation unit	70 (25)	9 (22)	15 (25)	19 (20)	27 (32)
Died	43 (15)	8 (20)	12 (20)	14 (15)	9 (11)
Aged care	6 (2)	0	0	5 (5)	1 (1)

Data are presented as number (percentage) or mean (standard deviation). Hyphen indicates data not available. APACHE, Acute Physiology and Chronic Health Evaluation; ICU, intensive care unit; SD, standard deviation.

The median hospital length of stay and hospital stay after ICU discharge both decreased over the study period (34.5 [26 to 53] versus 42 [29 to 73] days, *P *= 0.06, and 19 [10 to 34] versus 30 [13 to 52] days, *P *< 0.05, for 2006 versus 2003, respectively). Although the distributions by year were not statistically different, the trend in hospital length of stay and hospital stay after ICU discharge were both statistically significant (*P *< 0.05 for both). The median time to tracheostomy insertion was 5 (3 to 7) days and this was unchanged over the 4 years. Median time from tracheostomy insertion to ICU discharge was 5 (3 to 9) days and was similar over the years of the study. There was a significant trend in the proportion of patients being discharged under the DRG high trim point of 43 days over time (*P *< 0.05). Of those patients who were decannulated, a higher proportion were discharged under the upper DRG trim point of 43 days over the 4 years of the study (*P *< 0.05). There was a significant trend to reduced decannulation times from ICU discharge (*P *< 0.01), although absolute differences between the years did not meet the criteria for statistical significance (*P *= 0.06) (Table 2). There was no statistical difference in time to tracheostomy, decannulation times, hospital or ICU lengths of stay, mortality rates, or discharge destination between patients with surgical and percutaneous tracheostomies.

**Table 2:** Outcomes for patients by year

	All	2003	2004	2005	2006
Hospital length of stay^a^	39 (26–60.5)	42 (29–73)	45 (27–65)	40 (25–59)	34.5 (26–53)
Length of stay after ICU^a^	21 (11.5–40)	30 (13–52)	25.5 (12.5–40)	20 (11–40)	19 (10–34)
					
ICU length of stay	11 (7.5–16)	10 (7–16)	11 (7.5–15)	10 (7–17)	11 (8–16)
Time to tracheostomy	5 (3–7)	-	5 (3–8)	5 (4–7)	5 (4–6)
					
Decannulation time^b^	9 (4–20)	14 (7–31)	9 (4.5–26)	10 (4–20)	7 (3–17)
Decannulation to discharge	12 (5–20)	12 (6–20)	13 (7–20)	12.5 (5–24)	9 (3.5–18.5)
					
Decannulated	241 (86)	33 (80)	48 (80)	80 (84)	80 (95)
Not decannulated	39 (14)	8 (20)	13 (20)	15 (16)	4 (5)
					
Discharge less than 43 days^a^	156 (55.7)	21 (51)	28 (47)	52 (55)	55 (66)
Decannulated and less than 43 days^a^	131 (54)	16 (49)	20 (42)	42 (53)	53 (66)

Data are presented as median time in days (interquartile range) or as number (percentage). Hyphen indicates data not available. ^a^*P *< 0.05 for trend; ^b^*P *< 0.01 for trend. ICU, intensive care unit.

**Figure 2:**
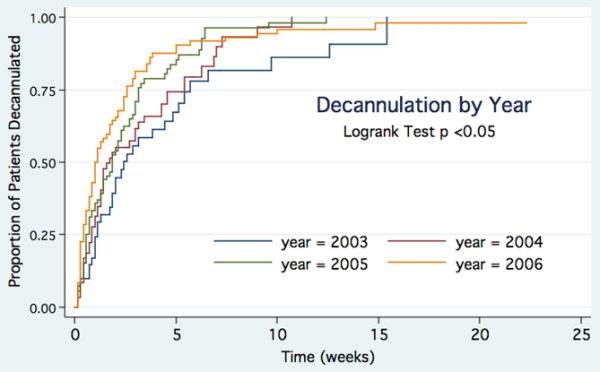
Kaplan-Meier plot of decannulation by year.

Crude decannulation rates per year increased over time with the rate ratio increasing by approximately 20% per year (1.2 [1.1 to 1.4]; *P *< 0.01 for trend). A greater proportion of patients were decannulated over successive years (*P *< 0.05). The log-rank test for equality of survivor functions for tracheostomies demonstrated significant differences between the years (*P *= 0.02) (Figure 2). Univariable analysis demonstrated that decannulation was related to year, admission unit, reason for admission, and tracheostomy indication (Table 3). Multivariable analysis showed that the hazard for decannulation increased by 25% (3% to 50%) per year. Compared with patients who had tracheostomies for prolonged ventilation/weaning, the hazard was decreased by 50% (25% to 66%) amongst patients whose reason for insertion was coma and was increased 2.1 (1.3 to 3.2) times if the indication was failed extubation. Compared with patients under medical units, the hazard was 52% (10% to 110%) higher for patients under the cardiothoracic service. There was no graphical or statistical evidence of violation of the proportional hazards assumption for the model (*P *= 0.34 for test of the proportional hazards assumption using Schoenfeld residuals).

**Table 3:** Univariate and multivariable analysis of decannulation

	Variable	Hazard ratio	*P *value	95% CI
Univariate	Year	1.22	<0.01	1.08–1.37
	Cardiothoracic unit	1.76	<0.01	1.26–2.44
	Neurosurgery unit	0.62	<0.01	0.44–0.85
	Cardiac surgery admission	1.71	<0.01	1.18–2.46
	CNS admission	0.58	<0.01	0.40–0.84
	Coma as indication	0.48	<0.001	0.33–0.70
	Failed extubation	2.0	<0.01	1.29–3.1
				
Multivariate	Year	1.25	0.02	1.03–1.49
	Coma as indication	0.5	<0.01	0.34–0.75
	Failed extubation	2.05	<0.01	1.33–3.16
	Cardiothoracic unit	1.52	0.01	1.11–2.1

CI, confidence interval; CNS, central nervous system.

## Discussion

This study suggests that, for patients discharged from the ICU with a tracheostomy, provision of tracheostomy care by an intensivist-led multidisciplinary team may lead to improvements in decannulation rates and length of stay. The principal reason for formation of a specialised tracheostomy review service was to improve care of patients discharged from the ICU with a tracheostomy. As one of the problems highlighted by allied health professionals prior to the formation of the team was the difficulty in obtaining medical reviews and delayed decision making regarding decannulation, it was felt that decannulation times would be a suitable outcome measure. This study shows that decannulation rates have improved and the improvements are independent of other variables such as indication and unit. There appears to be a learning effect for the intervention with outcomes improving over time. There are few comparative data on average decannulation times but a paper from a tertiary referral hospital in the same city had considerably longer median decannulation times for their ICU patients discharged to the ward with a tracheostomy (25 [19 to 34] days versus 9 [4 to 20] days) [8].

The mechanism by which a tracheostomy team might improve decannulation and admission times is likely to be multifactorial. Review by experienced people may reduce tracheostomy complications that delay recovery whilst a multidisciplinary team allows consensus decisions regarding tracheostomy weaning and decannulation to be made and enacted without the delays associated with multiple separate reviews. Having a senior medical practitioner as part of the team is important in this respect as it provides an auspice of authority under which nurse and allied health professionals can act without the usual delays of consulting the parent team. Other elements of the service which may influence outcomes are education and support of ward staff [9]. ICU liaison nurse programs are associated with benefits in terms of ICU readmissions, mortality, and morbidity [10,11]. It is thus possible that the regular review of patients by a liaison nurse may have improved outcomes independently of tracheostomy care.

A major limitation of this study was the retrospective nature of data collection for the period prior to the formation of the team. This limited the nature of data available for comparison and raises the possibility that there were other factors influencing tracheostomy care – either positively or negatively – which we are not aware of. The MET system was in place for both periods, suggesting that this is unlikely to be a factor, but ICU liaison nurse services began in 2006 and this may have had some impact on results. We are unaware of any other significant changes in ward care over this time. Whilst a cohort study such as this cannot prove that the intervention was responsible for the change, the temporal change over a short time period is supportive of the assumption of cause and effect.

Length of stay was one of the secondary outcome measures of interest. The DRG for tracheostomy is the third highest ranking DRG in terms of bed days occupied in public hospitals in Australia and is responsible for the highest cost by volume of any DRG [12]. Discharge below the high trim point of 43 days may result in financial savings for the hospital. We were able to demonstrate a significant trend in the reduction of hospital length of stay and length of stay after ICU discharge and in the proportion of patients being discharged below the DRG high trim point.

A tracheostomy service cannot influence hospital stay prior to ICU admission nor is it likely to greatly influence stay after decannulation where underlying medical problems and discharge processes are the major determinants. There are few discharge options for patients with tracheostomies as the majority of rehabilitation facilities and district hospitals are unwilling to accept such patients and as a result discharge planning is often delayed until its removal. Better discharge planning based on team estimates as to when tracheostomy is likely to be removed and a willingness of rehabilitation services to see patients prior to tracheostomy removal might further shorten hospital length of stay.

The proportion of patients decannulated increased over the study period, which may reflect a more proactive approach to decannulation. Decannulation is now sometimes performed as part of the palliative care process to allow a more natural and dignified death for the patient and their family. Only one patient died with a tracheostomy *in situ *in 2006 compared with 5 in 2003, 11 in 2004, and 9 in 2005.

Discharge outcomes for tracheostomy patients reflect the severity and complexity of the underlying disease processes. Few patients (18%) were discharged directly home; the majority were discharged to rehabilitation units or other hospitals. This is compared with the 43% of patients being discharged home in the multicentre study of ICU tracheostomy patients by Frutos-Vivar and colleagues [13]. In that study, the indications for tracheostomy were similar to our series; however, the patients were younger, which may explain the differences in discharge destination. In-hospital mortality rates at our institution are similar to those reported in the literature for ICU patients discharged to the ward with a tracheostomy. Overall mortality in this series is 15.4%, which is very similar to that reported by Clec'h and colleagues [7] (15.25%) and Flaatten and colleagues [6] (15.9%). Mortality in our series tended to decrease over time, with mortality being 10.7% in 2006, although this trend was not statistically significant.

There is little in the literature on tracheostomy management following ICU discharge. Krishnan and colleagues [14] reported that, in 75% of units in the UK responding to a postal service, ICU physicians or outreach nurses undertook decannulation but only a quarter had a written protocol for post-discharge tracheostomy care. Norwood and colleagues [15] reported results of a physiotherapist-led team that attempted to remove tracheostomies in the ICU prior to discharge and used mini-tracheostomies wherever possible for patients requiring suctioning following discharge from the ICU. The authors were able to show a reduction in patients discharged to the ward with a tracheostomy *in situ *and in complications on the ward. Whilst attempting to decannulate patients prior to ICU discharge may improve patient care, such a practice would require increased time in the ICU (not reported in the study), something our ICU could not provide due to pressure on beds.

This service was implemented without additional funding or staff. Initially, the ICU research nurse accompanied the round and was responsible for data collection and entry, but with the introduction of a liaison nurse position, the role became a liaison activity. The involved intensivist is a full-time employee and rounds were incorporated into standard clinical duties. For the physiotherapist, speech pathologist, and dietician, there was no increase in staffing levels, resulting in an increase of about 4 hours of clinical duties each per week. Funding for allied health members remains an issue.

## Conclusion

The institution of a tracheostomy team to manage tracheostomy care of patients discharged from the ICU with a tracheostomy was associated with improvements in decannulation rates and in length of stay. As well as improving patient care, services such as this may result in cost savings for the health service.

## Key messages

• With the advent of percutaneous tracheostomy, patients may be discharged from the intensive care unit (ICU) to the wards without formalised follow-up by medical staff with specialist tracheostomy knowledge.

• The effect of an intensivist-led multidisciplinary team to oversee ward management and decannulation of such patients is described.

• Compared with outcomes prior to the intervention, time to decannulation and length of hospital stay after ICU discharge decreased.

• An intensivist-led tracheostomy team is associated with improved outcomes and may potentially lead to financial savings for the health service.

## Abbreviations

APACHE II = Acute Physiology and Chronic Health Evaluation II; DRG = disease-related group; ENT = ear, nose, and throat; ICU = intensive care unit; MET = medical emergency team.

## Competing interests

The authors declare that they have no competing interests.

## Authors' contributions

AT was the intensivist in charge of the tracheostomy service and was responsible for data cleaning, initial statistical analysis, and drafting the manuscript. JS provided statistical analysis and assisted in drafting of the manuscript. Both authors read and approved the final manuscript.
